# Assessment of Dental Anxiety in a Young Population of the North Indian City of Lucknow

**DOI:** 10.7759/cureus.72977

**Published:** 2024-11-04

**Authors:** Arushi Singh, Monika Rathore, Bibhav Dubey

**Affiliations:** 1 Psychology, Vinay Neuropsychiatry Clinic, Lucknow, IND; 2 Pedodontics and Preventive Dentistry, Babu Banarasi Das College of Dental Sciences, Babu Banarasi Das University, Lucknow, IND

**Keywords:** anxiety management, behaviour, dental anxiety, dental fear, gender difference, mdas, oral health, psychology, young adults

## Abstract

Introduction: Dental anxiety is a common fear, especially among younger age groups, and it can prevent individuals from seeking necessary dental treatment. Effective communication between dental professionals and young adults is crucial to address this issue. However, factors such as past experiences, peer influences, and coping strategies can impact a young adult's anxiety in a dental setting. This study aims to assess levels of dental anxiety among young adults in Lucknow using the Modified Dental Anxiety Scale (MDAS).

Methodology: A total of 137 participants (76 females, 61 males), aged 15-20 years, were surveyed across various dental clinics in Lucknow. Statistical analysis using IBM SPSS Statistics for Windows, Version 23 (Released 2015; IBM Corp., Armonk, New York) was conducted to evaluate differences in dental anxiety levels between genders.

Result: The results revealed a significant difference in anxiety levels between males and females in specific situations, such as sitting in a waiting room, having a tooth drilled, and receiving local anaesthesia, with females exhibiting higher anxiety scores. However, no significant difference was observed when participants were asked about visiting a dentist the next day or undergoing scaling and polishing.

Conclusion: The study concludes that dental anxiety is prevalent among young adults in Lucknow, with females showing higher anxiety levels than males in most situations. Understanding these patterns can help dental professionals tailor their approaches to managing anxiety in younger patients.

## Introduction

The fear of dental treatment is one of the most significant concerns individuals experience, often preventing people, particularly younger age groups, from seeking necessary dental care [1]. Establishing effective communication between dental professionals and young adults can help to reduce their anxiety during treatment. Despite the best efforts of dental teams, including pre-treatment discussions, behavioural management techniques such as cognitive behavioural therapy (CBT) and relaxation strategies, as well as options for sedation and analgesia, fears remain, making routine dental procedures challenging [2]. Several factors impact a young adult's response in a dental setting, including cognitive development, coping mechanisms, peer influences, and previous dental or medical experiences [2].

Human development is a multidimensional and lifelong process involving changes in size, shape, function, structure, and skill. Psychological development occurs along a continuum rather than in distinct stages, meaning that experiences accumulate and interact over time [3]. Experiences during childhood and adolescence, in particular, play a pivotal role in shaping emotional responses and behaviours that carry into adulthood. Fear, including fear of dental treatment, often stems from these early developmental periods.

Learning and behavioural theories highlight how environmental factors, such as past traumatic experiences or exposure to negative stimuli (e.g., painful procedures or distressing dental visits), can shape an individual’s behaviour. By influencing how individuals perceive and respond to certain situations, these environmental factors contribute to the development of anxiety or fear. Thus, when clinicians can identify the primary causes of fear, especially those rooted in developmental experiences, they can effectively tailor interventions to alleviate anxiety. Addressing these fears early on and understanding their developmental origins are crucial for better management and prevention.

Young adults face various stressors in their daily lives, with dental treatment being one of the most common sources of fear. Both children and adults experience this anxiety, and stressful dental experiences in early life can result in a lifelong fear of dental treatment [3]. The present study aims to understand the dental fears of young adults and develop strategies to address them in clinical settings.

Several anxiety scales have been employed and trusted by researchers over time. One such tool is the Modified Dental Anxiety Scale (MDAS), developed by Professor Gerry Humphris in 1995 at the University of St. Andrews in Scotland [4]. It was based on Corah’s Dental Anxiety Scale (DAS) [5], with modifications to simplify the language for patients. A fifth question regarding anxiety about needle injections was added as a common trigger. The MDAS features shorter, easier-to-understand questions, making it widely used for assessing dental anxiety.

The null hypothesis (H₀) of this study is: "There is no significant difference in dental anxiety levels between male and female respondents as assessed by the MDAS." This study aimed to evaluate dental anxiety in young adults of both genders in the northern Indian city of Lucknow.

## Materials and methods

This study was conducted across 10 private dental clinics in Lucknow. A convenience sample of 137 participants was selected over three months based on the following criteria.

Inclusion criteria

Physically and mentally healthy adolescents aged 15 to 20 years, from both genders, were recruited. Participants had not undergone any dental procedures other than routine checkups and oral hygiene treatments.

Exclusion criteria

Individuals with significant mental abnormalities or other diagnosable neurological disorders were excluded from the study. Participants for whom consent could not be obtained were also excluded.

Participants were selected using a non-random sampling method. The sample consisted of 137 respondents (76 females, 61 males) aged 15-20 years. Participants' physical and mental health was screened via a self-reported health history questionnaire, which included information on existing medical conditions, mental health diagnoses, and ongoing treatments, to ensure appropriate inclusion or exclusion. These individuals were either accompanying a family member for dental treatment or seeking treatment themselves at the dental clinic. Randomization was achieved by enrolling participants without preselection based on their reason for visiting the clinic. After explaining the study’s purpose and obtaining informed consent or assent, participants who agreed to take part were given the MDAS (Table 1) questionnaire to complete. Immediate, on-site data collection minimised potential biases from premeditated responses or external influences, thereby ensuring randomness in participation. The completed questionnaires were evaluated, and the data were tabulated by the principal investigator. These data were then subjected to statistical analysis, from which inferences were drawn.

**Table 1:** Modified Dental Anxiety Scale

Questions	1. Not anxious	2. Slightly anxious	3. Fairly anxious	4. Very anxious	5. Extremely anxious
Q1: How would you feel if you went to your dentist tomorrow?					
Q2: How would you feel if you were sitting in the waiting room?					
Q3: How would you feel if you were about to have a tooth drilled?					
Q4: How would you feel if you were about to have your teeth scaled and polished?					
Q5: How would you feel if you were about to receive a local anaesthetic injection?					

Instruments

The MDAS was used to quantify participants' levels of dental anxiety. The MDAS consists of five multiple-choice questions, each with a 5-point rating scale, ranging from "not anxious" to "extremely anxious" (Table 1). Each question carries a minimum possible score of 1 and a maximum score of 5, resulting in a total possible score range of 5 to 25 for the entire scale [6].

Data analysis

The collected responses were entered into Microsoft Excel (Microsoft Corporation, Redmond, Washington) and analysed using IBM SPSS Statistics for Windows, Version 23 (Released 2015; IBM Corp., Armonk, New York). Mean MDAS scores were calculated for all participants. Student’s t-test was conducted to evaluate differences in anxiety scores between genders, with p-values as follows: p < 0.05 (*) considered statistically significant, p < 0.001 (**) indicating a highly significant difference, and NS standing for not significant.

## Results

The study surveyed a total of 137 participants, comprising 61 males (44.52%) and 76 females (55.47%), within the age group of 15-20 years (Table 2). These demographic characteristics ensure that the sample is representative of the general population attending various dental clinics across Lucknow, allowing for a balanced analysis of dental anxiety levels across genders and age groups.

**Table 2:** Demographic information of participants. N: number of participants.

Demographic characteristics	N	Percentage (%)
Total participants	137	100%
Gender		
Male	61	44.52%
Female	76	55.47%

Regarding anxiety levels for different dental procedures (Table 3), Q1 assessed anxiety about visiting the dentist the next day, revealing no significant difference between males (mean = 2.21 ± 1.39) and females (mean = 2.17 ± 0.74), with a p-value of 0.051. This suggests that the anticipation of a dental visit elicits similar levels of mild anxiety in both genders. In Q2, concerning anxiety in the waiting room, a significant difference (p < 0.05) was observed, with females (mean = 3.22 ± 1.01) reporting higher anxiety than males (mean = 2.84 ± 1.23), indicating that females tend to experience more anxiety while awaiting dental treatment. Q3 evaluated anxiety about having a tooth drilled and found a highly significant difference (p < 0.001), with females (mean = 3.70 ± 0.94) displaying greater anxiety than males (mean = 3.02 ± 1.15). This suggests that invasive procedures, such as tooth drilling, provoke stronger anxiety responses, particularly among females. For Q4, assessing anxiety about scaling and polishing, no significant difference was found between males (mean = 2.33 ± 1.06) and females (mean = 2.38 ± 0.88), with a p-value of 0.105, suggesting that both genders experience similar levels of mild anxiety for routine cleaning procedures. However, in Q5, anxiety regarding receiving a local anaesthetic injection showed a highly significant difference (p < 0.001), with females (mean = 3.98 ± 1.01) reporting much higher anxiety than males (mean = 2.66 ± 1.42). This indicates that fear of injections is a significant source of anxiety for females in dental settings. Overall, these findings reveal that, while the general anticipation of a dental visit does not differ significantly between genders, females consistently exhibit higher anxiety in more specific and invasive situations, particularly when sitting in the waiting room, undergoing tooth drilling, or receiving local anaesthetic injections. These results suggest that gender plays a crucial role in dental anxiety, especially regarding invasive procedures.

**Table 3:** Gender-based anxiety levels for different dental procedures. N: number of participants, SD: standard deviation, Std. Error: standard error of the mean values. p < 0.05 was considered statistically significant (*); p < 0.001 (**) indicates a highly significant difference; NS not significant.

Questions	Gender	N	Mean	SD	Std. Error	95% Confidence interval	t-test	P-value	Significance level
						Lower bound	Upper bound		
Q1: How would you feel if you went to your dentist tomorrow?	Male	61	2.21	1.39	0.1782	1.87	2.57	0.051	NS
	Female	76	2.17	0.74	0.0855	2	2.34		
Q2: How would you feel if you were sitting in the waiting room?	Male	61	2.84	1.23	0.1571	2.53	3.15	4.096	p < 0.05
	Female	76	3.22	1.01	0.1164	2.99	3.46		
Q3: How would you feel if you were about to have a tooth drilled?	Male	61	3.02	1.15	0.1469	2.72	3.31	18.64	p < 0.001
	Female	76	3.7	0.94	0.1076	3.49	3.91		
Q4: How would you feel if you were about to have your teeth scaled and polished?	Male	61	2.33	1.06	0.1355	2.06	2.6	0.105	NS
	Female	76	2.38	0.88	0.1002	2.18	2.59		
Q5: How would you feel if you were about to receive a local anaesthetic injection?	Male	61	2.66	1.42	0.1824	2.29	3.02	40.133	p < 0.001
	Female	76	3.98	1.01	0.1154	3.74	4.2		

**Figure 1:**
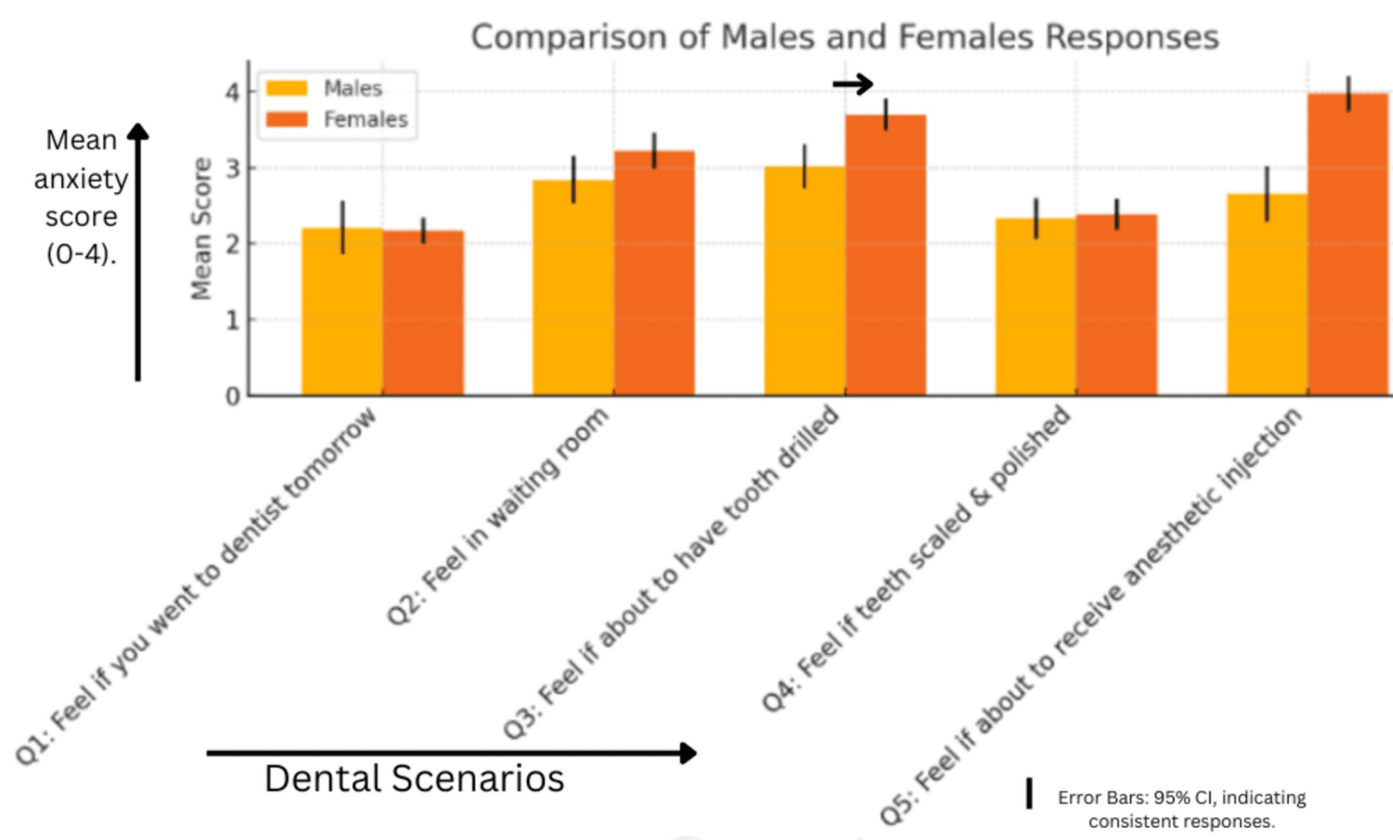
Graphical representation of gender-based anxiety levels for different dental procedures.

The graph compares male and female responses to five dental scenarios, measured on a 1 to 5 anxiety scale. The x-axis represents different dental situations (e.g., visiting the dentist, sitting in the waiting room, tooth drilling, scaling, and receiving an anaesthetic injection), while the y-axis displays mean anxiety scores (Figure 1). Males consistently report lower anxiety, particularly for tooth drilling and anaesthetic injections, compared to females. Females show significantly higher mean scores in these scenarios (Q3 and Q5), indicating greater discomfort or anxiety. Error bars represent 95% confidence intervals, which are relatively narrow, suggesting consistent responses across participants for both genders.

This outcome leads to the rejection of the null hypothesis, indicating that females generally experience higher anxiety than males regarding dental treatments.

## Discussion

Dental fear and anxiety are prevalent across all ages, with evidence indicating that fears developed during childhood and adolescence often persist into adulthood [7, 8]. A well-planned approach to managing anxiety in children and adolescents is essential for fostering a positive attitude towards dental treatments as they grow. Understanding the causes and levels of anxiety in this population is crucial for professionals to address these concerns effectively.

Implications and future recommendations

The findings of this study underscore the need for dental professionals to develop targeted strategies that cater to the unique needs of young patients. For instance, it is essential to implement anxiety management techniques specifically designed for adolescents and young adults, who may be less likely to express their fears openly. Future research should focus on developing interventions that encourage these age groups to communicate their anxieties, facilitating better management of their dental experiences.

Additionally, it is important to consider the influence of various factors on dental anxiety. Practitioners should take into account not only demographic variables but also personal histories, such as previous dental experiences and cultural backgrounds. Understanding these influences can lead to more individualized care plans that effectively reduce anxiety in young patients. Children typically express their fears more clearly, enabling caregivers to understand and address their concerns effectively [9]. In contrast, adolescents and young adults, who possess a greater sense of autonomy, may conceal their emotions to avoid appearing vulnerable [9]. Consequently, these genuine fears are less likely to be revealed and adequately addressed.

Another area of concern is the common perception that males are less anxious and fearful than females during stressful situations, such as dental visits. This study aimed to explore these fears in both male and female participants [10]. The MDAS was used due to its practicality in outpatient dental clinic settings.

Our results indicate that neither gender exhibited significant anxiety the day before their dental checkup (Table 3). Anxiety towards scaling and polishing was also similar between genders, possibly because these procedures are often perceived as aesthetic rather than therapeutic by the general public.

Interestingly, females consistently displayed higher anxiety scores across various parameters compared to their male counterparts. This observation aligns with existing research, which suggests that women tend to report higher levels of dental fear and anxiety in stressful situations such as dental visits [11-14]. Factors such as greater emotional expressiveness, societal expectations, and biological responses to stress may contribute to this gender difference [12-15]. Conversely, males often report lower anxiety levels, potentially influenced by cultural norms that discourage the expression of fear or discomfort [10-15]. Our findings reveal a significant prevalence of dental anxiety among young individuals, consistent with results reported by Mohammed et al., which also indicated heightened anxiety levels in a comparable age group [16].

The present study has several limitations that should be acknowledged. First, the reliance on self-report measures may introduce bias, as participants might underreport their anxiety due to social desirability or a lack of self-awareness. Furthermore, the relatively small sample size from a single geographic location may limit the generalizability of the findings to the broader population. Additionally, other confounding variables, such as previous dental experiences and cultural influences, were not analysed, highlighting the need for further research to gain comprehensive insights into dental anxiety in young adults.

## Conclusions

The overall anxiety towards dental procedures was high in both genders, indicating that appropriate measures should be taken to reduce dental anxiety in young adults for the sake of their oral health. Effective strategies and interventions are crucial to help alleviate these fears and ensure that young adults develop a more positive attitude towards dental care.

In this study, female respondents consistently showed higher dental anxiety than their male counterparts. This highlights the need for gender-specific approaches to addressing dental anxiety. By understanding and addressing these differences, dental professionals can create a more supportive and anxiety-free environment for both male and female patients.
